# The structure of the complex between the arsenite oxidase from *Pseudorhizobium banfieldiae* sp. strain NT-26 and its native electron acceptor cytochrome *c*_552_

**DOI:** 10.1107/S2059798323002103

**Published:** 2023-03-30

**Authors:** Nilakhi Poddar, Joanne M. Santini, Megan J. Maher

**Affiliations:** a School of Chemistry and The Bio21 Molecular Science and Biotechnology Institute, The University of Melbourne, Parkville, Australia; b Institute of Structural and Molecular Biology, Division of Biosciences, University College London, London WC1E 6BT, United Kingdom; University of Western Australia, Crawley, Australia

**Keywords:** electron transfer complexes, X-ray crystallography, arsenite, molybdenum enzymes, *Pseudorhizobium banfieldiae* sp. strain NT-26, cytochrome *c*_552_

## Abstract

The arsenite oxidase (AioAB) from *Pseudorhizobium banfieldiae* sp. strain NT-26 catalyzes the oxidation of arsenite to arsenate and transfers electrons to its cognate electron acceptor cytochrome *c*_552_ (cyt*c*_552_). This activity underpins the ability of this organism to respire using arsenite present in contaminated environments. The crystal structure of the AioAB/cyt*c*_552_ electron transfer complex reveals two A_2_B_2_/(cyt*c*_552_)_2_ assemblies per asymmetric unit. Three of the four cyt*c*_552_ molecules in the asymmetric unit dock to AioAB in a cleft at the interface between the AioA and AioB subunits, with an edge-to-edge distance of 7.5 Å between the heme of cyt*c*_552_ and the [2Fe–2S] Rieske cluster in the AioB subunit. The interface between the AioAB and cyt*c*_552_ proteins features electrostatic and nonpolar interactions and is stabilized by two salt bridges. A modest number of hydrogen bonds, salt bridges and relatively small, buried surface areas between protein partners are typical features of transient electron transfer complexes. Interestingly, the fourth cyt*c*_552_ molecule is positioned differently between two AioAB heterodimers, with distances between its heme and the AioAB redox active cofactors that are outside the acceptable range for fast electron transfer. This unique cyt*c*_552_ molecule appears to be positioned to facilitate crystal packing rather than reflecting a functional complex.

## Abstract 2

The crystal structure of the electron transfer complex between arsenite oxidase (AioAB) from *Pseudorhizobium banfieldiae* sp. strain NT-26 and its native electron acceptor cytochrome *c*_552_ (cyt*c*_552_) is presented. Cyt*c*_552_ docks within a cleft at the interface of the AioA and AioB subunits, which allows a close association between redox cofactors.; this close association presumably facilitates fast electron transfer and underpins the ability of this organism to respire in arsenic contaminated environments.

## 1. Introduction

Interprotein electron transfer processes are key biochemical events which play critical roles in fundamental biological processes such as photosynthesis, respiration and nitrogen fixation (Berg *et al.*, 2007 ▸). The structural characterization of protein–protein complexes that participate in electron transfer is challenging due to their weak and transient nature (Supplementary Table S1; Antonyuk *et al.*, 2013 ▸). The formation of electron transfer complexes requires efficient and finely tuned docking and dynamics at the protein–protein interface (Bendall, 2020 ▸; Moser *et al.*, 1992 ▸), with complexes being guided together by complementary electrostatic steering and the relative positions of the proteins being tuned through hydrophobic/van der Waals interactions (Leys & Scrutton, 2004 ▸). The distance between the redox centers within these complexes has been shown to influence the rate of electron transfer (Davidson, 2000 ▸; Marcus & Sutin, 1985 ▸; Moser *et al.*, 1992 ▸).

Interprotein electron transfer processes are crucial for the survival of arsenic respiring organisms cultured from arsenic contaminated environments (Santini *et al.*, 2007 ▸). Arsenic is toxic to most organisms in its inorganic forms arsenite ( 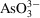 ) and arsenate ( 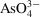 ) (Bissen & Frimmel, 2003 ▸; Domingo, 1995 ▸); however, prokaryotes such as *Pseudorhizobium banfieldiae* sp. strain NT-26 can catalyze the aerobic oxidation of arsenite (to arsenate) through the action of the enzyme arsenite oxidase (AioAB). The physiological electron acceptor for the AioAB enzyme has been shown to be a soluble *c*-type cytochrome (cyt*c*_552_; Santini *et al.*, 2007 ▸; Santini & vanden Hoven, 2004 ▸).

The AioAB complex consists of two subunits: a large subunit, AioA (93 kDa), which contains a molybdenum cofactor (Moco) at the active site and a [3Fe–4S] cluster, and a small subunit, AioB (14 kDa), which contains a Rieske [2Fe–2S] cluster. AioAB is a member of the dimethyl sulfoxide (DMSO) reductase family of molybdoenzymes. The crystal structure of AioAB from *P. banfieldiae* sp. strain NT-26 has been determined and refined to 2.7 Å resolution (PDB 4aay; Warelow *et al.*, 2013 ▸). The proposed reaction mechanism of AioAB comprises oxidation of arsenite at the molybdenum site [reducing molybdenum(VI) to molybdenum(IV)], which releases two electrons that transfer one at a time to the [3Fe–4S] cluster of the AioA subunit and then to the [2Fe–2S] Rieske cluster of the AioB subunit. The electrons are then received by the electron acceptor cyt*c*_552_ (Bernhardt & Santini, 2006 ▸; Santini & Ward, 2018 ▸; Supplementary Fig. S1).

To investigate the structural basis of the electron transfer process that underpins respiration using arsenite, here we report the crystal structure of the AioAB/cyt*c*_552_ complex from *P. banfieldiae* sp. strain NT-26. We show that cyt*c*_552_ sits within a cleft at the interface between the AioA and AioB subunits, with a relatively short distance between redox-active cofactors. The crystals show an interesting arrangement, with three of the four cyt*c*_552_ molecules located in a ‘functional’ location. The positioning of the fourth cyt*c*_552_ seems to be ‘nonfunctional’ and presumably facilitates crystal packing.

## 2. Materials and methods

The pPROEX-HTb-AioBA and pET-22b(+)-cyt*c*_552_ plasmids were prepared as described previously (Santini *et al.*, 2007 ▸; Warelow *et al.*, 2013 ▸). In these constructs the AioA subunit is composed of residues 2–845, the AioB subunit is composed of residues 41–175 and cyt*c*_552_ is composed of residues 21–127, in addition to residues derived from affinity tags (Supplementary Table S4). Residue numbering corresponds to the respective UniProt entries (Q6VAL8, Q6VAL9 and Q2TV05).

### 2.1. Protein overexpression and purification

The pPROEX-HTb-AioBA plasmid was transformed into *Escherichia coli* strain DH5α (New England Biolabs). Cultures were grown aerobically at 21°C in Luria broth (LB) with 1 m*M* sodium molybdate and supplemented with ampicillin (100 µg ml^−1^) with slow shaking (∼50 rev min^−1^). The cultures were induced with 40 µ*M* isopropyl β-d-thiogalactopyranoside (IPTG) and were harvested after 24 h.

His-AioBA was purified by immobilized metal-affinity chromatography (IMAC) followed by size-exclusion chromatography (SEC). Frozen cell pellets were thawed and resuspended in binding buffer (20 m*M* potassium phosphate, 500 m*M* NaCl, 20 m*M* imidazole pH 7.3). The cells were lysed using a TS series bench-top cell disruptor (Constant Systems) at 241 MPa and insoluble debris was removed by centrifugation (Beckman JLA-25.50; 30 000*g*, 1 h, 4°C). The soluble fraction was incubated with 5 ml Ni-Sepharose 6 Fast Flow resin (Cytiva; 4°C; 1 h stirring) that had been pre-equilibrated with binding buffer (20 m*M* potassium phosphate, 500 m*M* NaCl, 20 m*M* imidazole pH 7.3). The resin was washed with ten column volumes (CV) of binding buffer, followed by elution of bound protein with elution buffer (20 m*M* potassium phosphate, 500 m*M* NaCl, 500 m*M* imidazole pH 7.3; 5 CV). The eluent was dialyzed (3000 molecular-weight cutoff SnakeSkin Dialysis Tubing, ThermoScientific) against 2.0 l dialysis buffer (50 m*M* MES pH 5.5) overnight. This resulted in the precipitation of contaminating proteins, which were removed by centrifugation (30 000*g*, 30 min). The supernatant was concentrated by centrifugal ultrafiltration (10 000 molecular weight cutoff, Millipore Amicon Ultra) and further purified by SEC (HiLoad 16/600 Superdex 200 pg, Cytiva; 4°C) in 50 m*M* MES, 100 m*M* NaCl pH 5.5. The purest fractions as determined by SDS–PAGE were pooled and concentrated to approximately 10 mg ml^−1^ by centrifugal ultrafiltration. Aliquots of the purified protein (which will be referred to as AioAB in the following) were snap-frozen and stored at −80°C until further use. The concentration of the AioAB enzyme was measured spectrophotometrically at 682 nm with ɛ_682_ = 5.6 m*M* ^−1^ cm^−1^ (Watson *et al.*, 2017 ▸).

The pET-22b(+)-cyt*c*_552_ and pEC86 plasmids were co-transformed into *E. coli* strain BL21(DE3) (New England Biolabs). Cultures were grown at 30°C in LB supplemented with ampicillin (100 µg ml^−1^), chloramphenicol (60 µg ml^−1^) and a 1:100 dilution of a trace-metal solution (Ihssen & Egli, 2004 ▸; Santini *et al.*, 2007 ▸). The cells were induced with 20 µ*M* IPTG at an OD_600_ value of between 1.2 and 1.5 and were harvested after 16 h of shaking at 30°C.

The His-cyt*c*_552_ protein was purified by cation-exchange chromatography, IMAC and SEC. Frozen cell pellets were thawed at room temperature and resuspended in cell-lysis buffer (20 m*M* MES pH 5.5). The cells were disrupted by passage through a TS series bench-top cell disruptor (Constant Systems) at 241 MPa. Cell debris was removed by centrifugation (Beckman JLA-25.50; 30 000*g*, 1 h, 4°C) and the soluble fraction was loaded onto a 5 ml HiTrap SP Sepharose Fast Flow column (Cytiva; 4°C), washed with buffer consisting of 70 m*M* NaCl, 20 m*M* MES pH 5.5 and eluted with a linear NaCl gradient (0.07–0.45 *M* in 50 m*M* MES pH 5.5). The eluent was dialyzed (3000 molecular weight cutoff SnakeSkin Dialysis Tubing, ThermoScientific) against 2.0 l dialysis buffer (50 m*M* potassium phosphate, 0.5 *M* NaCl pH 7.4) overnight. The protein was loaded onto a 5 ml HisTrap column (Cytiva) and eluted using an imidazole gradient (0–0.5 *M* in 50 m*M* potassium phosphate, 0.5 *M* NaCl pH 7.4) followed by SEC (HiLoad 16/600 Superdex 75 pg, Cytiva; 4°C; 20 m*M* Tris, 150 m*M* NaCl pH 7.8). The purified protein (which will be referred to as cyt*c*_552_) was concentrated to 10 mg ml^−1^ and stored at −80°C until further use. The concentration of oxidized cyt*c*_552_ was determined spectrophotometrically at 550 nm using ɛ_550_ = 8.7 m*M* ^−1^ cm^−1^ (Santini *et al.*, 2007 ▸).

### 2.2. Enzyme kinetics

AioAB activity assays were carried out as described previously (Watson *et al.*, 2017 ▸). The reduced–oxidized extinction coefficient for cytochrome *c*_552_ at 550 nm is 23 m*M* ^−1^ cm^−1^ (Santini *et al.*, 2007 ▸) and that at 416 nm is 59 m*M* ^−1^ cm^− 1^ (Santini *et al.*, 2007 ▸). Purified AioAB enzyme (2 n*M*) was incubated with fully oxidized cytochrome *c*_552_ (20 µ*M*) in 50 m*M* Tris–HCl pH 8.0 (Watson *et al.*, 2017 ▸) with increasing concentrations of arsenite (0–1 m*M*). The reaction was followed at 550 nm. The steady-state kinetics with cyt*c*_552_ as the substrate were determined using an excess of arsenite (2.5 m*M*) pre-incubated with purified AioAB enzyme (2 n*M*), followed by the addition of various concentrations of cyt*c*_552_ (0–10 µ*M*). In this case, the reaction was followed at 416 nm (Watson *et al.*, 2017 ▸). Kinetic experiments were performed using triplicate measurements and data fitting was carried out using the Michaelis–Menten function with *GraphPad Prism* version 7.0 for Mac OS X (GraphPad Software, La Jolla, California, USA).

### 2.3. Protein crystallization and data collection

Purified AioAB (in 50 m*M* Tris, 100 m*M* NaCl pH 7.8) and cyt*c*_552_ (in 50 m*M* Tris, 100 m*M* NaCl pH 7.8) were mixed and incubated on ice at a molar ratio of 1:1.5 AioAB:cyt*c*_552_ (total protein concentration 5 mg ml^−1^) before crystallization via sitting-drop vapor diffusion in 96-well plates (Molecular Dimensions). The stoichiometry of the mixture (AioAB:cyt*c*_552_) was based on our previous experience in crystallizing the SorT/SorU complex from *Sinorhizobium meliloti* (McGrath *et al.*, 2015 ▸). Initial crystallization trials for the AioAB/cyt*c*_552_ complex were conducted using the Index HT (Hampton Research) and ProPlex (Molecular Dimensions) screens. Drops consisting of equal volumes (0.2 µl) of reservoir solution and protein solution were dispensed by a Crystal Gryphon liquid-handling system (Art Robbins Instruments) and were equilibrated against a 50 µl reservoir of screen solution at 20°C. Multiple plate-like crystals of AioAB/cyt*c*_552_ were observed within one week in conditions A4 (0.1 *M* bis-Tris pH 6.5, 2.0 *M* ammonium sulfate), E7 [0.05 *M* magnesium chloride hexahydrate, 0.1 *M* HEPES pH 7.5, 30%(*v*/*v*) PEG 3350], F12 [0.2 *M* sodium chloride, 0.1 *M* HEPES pH 7.5, 25%(*w*/*v*) PEG 3350], G1 [0.2 *M* sodium chloride, 0.1 *M* Tris–HCl pH 8.5, 25%(*w*/*v*) PEG 3350], H1 [0.2 *M* magnesium chloride hexahydrate, 0.1 *M* Tris–HCl pH 8.5, 25%(*w*/*v*) PEG 3350] and H6 [0.2 *M* sodium formate, 20%(*w*/*v*) PEG 3350] of the Index HT screen. Crystals were also observed within one week in conditions C3 [0.2 *M* ammonium acetate, 0.1 *M* sodium citrate, 20%(*w*/*v*) PEG 4000], C8 [0.2 *M* sodium chloride, 0.1 *M* Tris–HCl pH 8.0, 20%(*w*/*v*) PEG 4000], C12 [0.2 *M* potassium iodide, 0.1 *M* MES pH 6.5, 25%(*w*/*v*) PEG 4000] and D1 [0.2 *M* sodium chloride, 0.1 *M* sodium HEPES pH 7.5, 25%(*w*/*v*) PEG 4000] of the ProPlex screen.

Optimization of these conditions was carried out by hanging-drop vapor diffusion in 24-well VDX plates (Hampton Research), varying the concentrations of sodium chloride (0.1–0.2 *M*), HEPES (0.05–0.1 *M*) and PEG 3350 (15–25%) and the pH (6.5–7.5). Diffraction-quality crystals of AioAB/cyt*c*_552_ grew after two weeks in drops consisting of equal volumes (2 µl; 1:1) of the AioAB/cyt*c*_552_ preparation and reservoir solution [0.2 *M* sodium chloride, 0.1 *M* HEPES pH 7.3, 18%(*w*/*v*) PEG 3350] equilibrated against 500 µl reservoir solution at 20°C. Crystals were cryoprotected in reservoir solution containing 25%(*w*/*v*) glycerol before flash-cooling in liquid nitrogen. Crystallization conditions are given in Table 1 ▸.

### 2.4. Data collection, structure solution and refinement

Diffraction data were collected from the AioAB/cyt*c*_552_ crystals using an EIGER 16M detector on beamline MX2 at 13 000 eV at the Australian Synchrotron. All data were collected at 100 K, processed with *XDS* (Kabsch, 2010 ▸) and merged and scaled with *AIMLESS* (Evans & Murshudov, 2013 ▸). Unit-cell parameters and data-collection statistics are presented in Table 2 ▸.

The crystal structure of the AioAB/cyt*c*_552_ complex was solved by molecular replacement with *MOLREP* (Vagin & Teplyakov, 2010 ▸) from the *CCP*4 suite (Winn *et al.*, 2011 ▸), using a search model composed of the coordinates of the AioAB structure (PDB entry 4aay; Warelow *et al.*, 2013 ▸) with the water molecules removed. Initial rounds of refinement of a model with four AioAB complexes per asymmetric unit yielded a difference Fourier electron-density map which showed positive difference density for the location of four molecules of cyt*c*_552_ per asymmetric unit. These were placed by phased molecular replacement with *MOLREP* using a search model generated from the structure of ferrocytochrome *c*_2_ (PDB entry 1co6; Badilla *et al.*, 2018 ▸) modified by *CHAINSAW* (Stein, 2008 ▸). Manual model building and the addition of water molecules were carried out in *Coot* (Emsley *et al.*, 2010 ▸) with iterative cycles of refinement using *REFMAC*5 (Murshudov *et al.*, 2011 ▸). The geometry of the final model was determined with *MolProbity *(Chen *et al.*, 2010 ▸). Refinement statistics are summarized in Table 3 ▸.

## 3. Results and discussion

The structure of the AioAB/cyt*c*_552_ complex was solved and refined to 2.25 Å resolution (Tables 2 ▸ and 3 ▸). The structure includes four copies of the AioAB assembly per asymmetric unit, arranged as two A_2_B_2_ heterotetramers. The AioA subunits include residues 2–844 and are composed of four domains (domains I, II, III and IV). Domain I is composed of three antiparallel β-sheets, domain II and domain III have similar αβα-sandwich topologies and domain IV predominantly consists of six antiparallel β-sheets flanked by five small α-helices. The AioB subunits include residues 44–175 and have a fold consisting of a six-stranded antiparallel β-barrel and a four-stranded antiparallel β-sheet.

The AioA subunit houses the Moco site, which is a common feature of the DMSO reductase family of molybdenum-containing enzymes, and the [3Fe–4S] cluster. The Mo atom is coordinated by one oxo ligand and the thiol groups of the two pterin cofactors in an approximate square-pyramidal geometry, with an average Mo=O distance across all four copies per asymmetric unit of 1.8 ± 0.1 Å. The [3Fe–4S] cluster is coordinated by a conserved cysteine-rich motif (Cys24-*X*_2_-Cys27-*X*_3_-Cys31-*X*_70_-Ser102) and the AioB subunit houses the [2Fe–2S] Rieske cluster, which is coordinated by two cysteine residues and two histidine residues (Cys103-*X*-His105-*X*_15_-Cys121-*X*_2_-His124).

In addition to the two AioA_2_B_2_ complexes, there are four molecules of cyt*c*_552_ per asymmetric unit. The cyt*c*_552_ protomers are composed of four α-helices arranged to form a bundle that frames a heme-binding site. His38 and Met103 are axial ligands of the central Fe atom and the porphyrin ring is covalently attached to Cys34 and Cys37 (Fig. 2*c* and Supplementary Fig. S6). This is the first reported crystal structure of cyt*c*_552_ from *P. banfieldiae* sp. strain NT-26. A search of the coordinates of cyt*c*_552_ against the Protein Data Bank (PDB) using *PDBeFold* (Krissinel & Henrick, 2004 ▸) reveals similarity to the structures of cyt*c*_552_ from *Paracoccus denitrificans* (PDB entry 1ql4; Harrenga *et al.*, 2000 ▸) and cyt*c*_2_ from *Rhodo­pseudomonas viridis* (PDB entry 1co6; Sogabe & Miki, 1995 ▸), with root-mean-square deviation (r.m.s.d.) values of 0.6–0.7 Å (over 92 and 79 common C^α^ positions), indicating similar structures.

### 3.1. Two different AioA_2_B_2_/(cyt*c*_552_)_2_ complexes are present in the crystal

**Figure 1:**
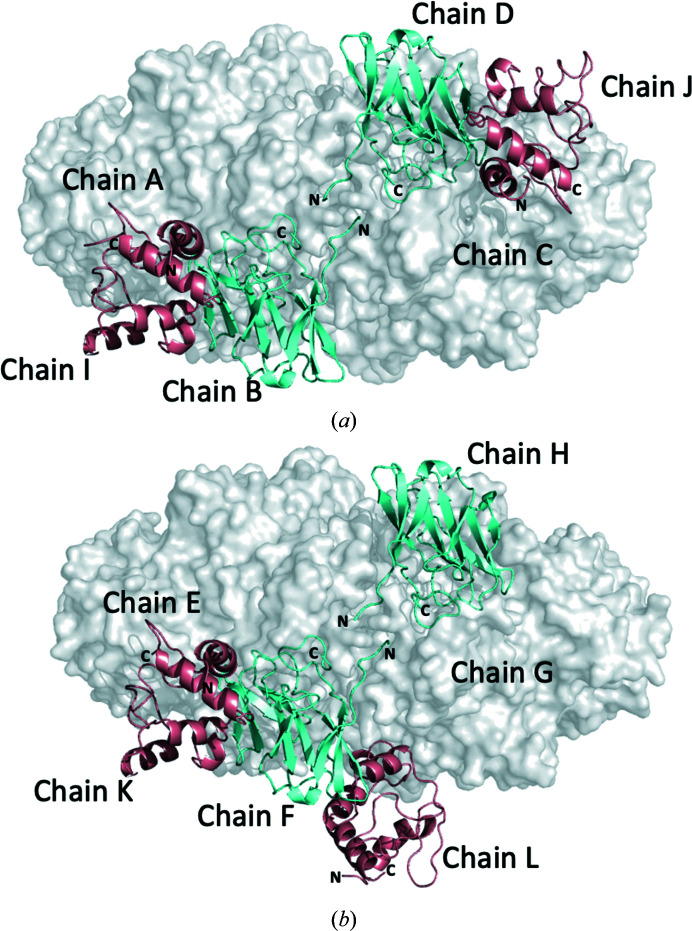
Two different AioA_2_B_2_/(cyt*c*_552_)_2_ complexes are present in the asymmetric unit of the AioAB/cyt*c*_552_ crystal structure. In both of the structures the AioA subunits are shown as gray surfaces, the cyt*c*_552_ molecules are in salmon and the AioB subunits are in cyan. (*a*) The AioA_2_B_2_ complex represented by chains *ABI* and *CDJ*. The two cyt*c*_552_ molecules (chains *I* and *J*) are located at similar positions in a cleft near the AioA/AioB interface. (*b*) The AioA_2_B_2_ complex represented by chains *EFK* and *GHL*. One molecule of cyt*c*_552_ (chain *K*) is located at the AioA/AioB interface. The second molecule of cyt*c*_552_ (chain *L*) is unique in that it sits between AioAB heterodimers.

In the asymmetric unit, two molecules of cyt*c*_552_ are associated with each of the two AioA_2_B_2_ assemblies, so there are two AioA_2_B_2_/(cyt*c*_552_)_2_ complexes per asymmetric unit (Fig. 1 ▸). In one complex (chains *ABI* and *CDJ*) the two cyt*c*_552_ molecules (chains *I* and *J*) are located at similar relative positions in a cleft near the AioA/AioB interface (Fig. 1 ▸ *a*). In the other AioA_2_B_2_/(cyt*c*_552_)_2_ unit (chains *EFK* and *GHL*) the relative positions of the two cyt*c*_552_ molecules are different. One (chain *K*) is consistent with that described above, sitting between the AioA and AioB subunits, while the other (chain *L*) associates with AioA (chain *G*) from one AioAB heterodimer and AioB (chain *F*) from the neighboring heterodimer (Fig. 1 ▸ *b*).

The three cyt*c*_552_ molecules that lie at the AioA/AioB interface (chains *I*, *J* and *K*) are located such that the edge-to-edge distance between the [2Fe–2S] Rieske cluster in AioB and the heme in cyt*c*_552_ is 7.5 Å, which is consistent with fast electron transfer (discussed further below; Page *et al.*, 1999 ▸). The unique cyt*c*_552_ (chain *L*) that associates between heterodimers shows edge-to-edge distances between the cyt*c*_552_ heme, the Moco (Mo atom) and the [3Fe–4S] cluster (residue Cys24) of AioA of 25 and 29 Å, respectively. The distance between the cyt*c*_552_ heme and the [2Fe–2S] cluster (residue Cys103) in AioB of the neighboring heterodimer is 38 Å (Supplementary Fig. S2). These distances are outside the accepted range for fast electron transfer. The positioning of the unique cyt*c*_552_ in the complex therefore does not represent an electron transfer complex. This positioning of cyt*c*_552_ presumably facilitates crystallization (Supplementary Fig. S3) but does not represent the complexes present in solution and/or a functional assembly. This is reminiscent of a previously reported structure of chicken liver sulfite oxidase (*Gallus gallus*; PDB entry 1sox; Kisker *et al.*, 1997 ▸). This enzyme contains three domains: an N-terminal cytochrome domain, a Moco domain and a C-terminal domain. Interestingly, in this structure the cytochrome domain is positioned so that the edge-to-edge distance is 32 Å between the Mo atom and the heme cofactor, which is also outside the range for fast electron transfer. In solution, a flexible linker between the Moco and cytochrome domains allows the cofactors to approach at proximity. The following discussion will therefore describe the AioAB/cyt*c*_552_ complex with cyt*c*_552_ positioned at the AioA/AioB interface.

### 3.2. The AioAB/cyt*c*_552_ interface

**Figure 2:**
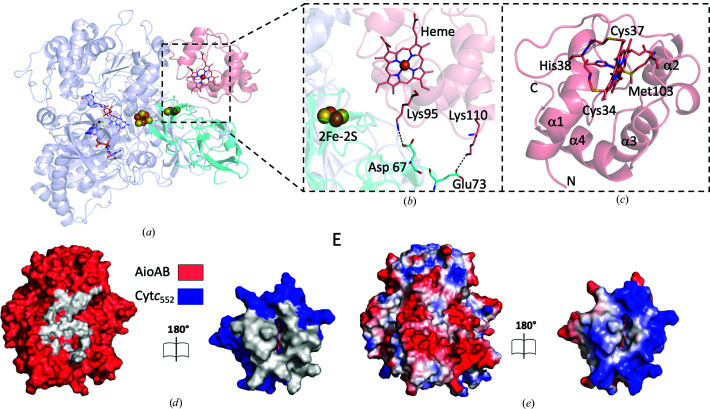
The structure of the AioAB/cyt*c*_552_ electron transfer complex. (*a*) The AioAB/cyt*c*_552_ complex as observed for chains *ABI*, *CDJ* and *EFK* (AioA in blue, AioB in cyan and cyt*c*_552_ in salmon). (*b*) The interface between AioAB and cyt*c*_552_. Residues that participate in the two salt bridges are shown. (*c*) The structure of cytochrome cyt*c*_552_. The four helices are labeled and the heme cofactor is shown in salmon. The heme Fe atom is coordinated by His38 and Met103. The protoporphyrin ring is covalently attached to Cys34 and Cys37. (*d*) ‘Open-book’ representation of the AioAB/cyt*c*_552_ complex (AioAB is in red and cyt*c*_552_ is in blue) indicating the ‘footprint’ of interacting residues for each protein. (*e*) The same view as in (*d*) colored according to the electrostatic surfaces (positive charge in blue, negative charge in red and neutral in white).

As described above, cyt*c*_552_ sits within a cleft near the AioA/AioB interface and interacts with both subunits of the AioAB heterodimer. Surface areas of 680 and 660 Å^2^ are buried on complex formation for the AioAB (350 Å^2^ for AioA and 330 Å^2^ for AioB) and cyt*c*_552_ proteins, respectively. The contacts between AioAB and cyt*c*_552_ are mediated by three regions of the cyt*c*_552_ structure (residues 32–37, 45–48 and 102–106; Fig. 2 ▸ *d*). Two salt bridges between Asp67 and Glu73 from the AioB subunit, and Lys95 and Lys110 from cyt*c*_552_ complete the interface (Figs. 2 ▸ *a* and 2 ▸ *b*). Notably, Asp67 and Glu73 from the AioB subunit are not conserved in the sequences of comparable Rieske proteins (Supplementary Fig. S5*a* ), whereas Lys95 of cyt*c*_552_ is conserved in the sequences of cyt*c*_552_ from *P. denitrificans* (*Pd*cyt*c*_552_; PDB entry 1ql4; Harrenga *et al.*, 2000 ▸) and cyt*c*_2_ from *R. viridis* (*Rv*cyt*c*_2_; PDB entry 1co6; Sogabe & Miki, 1995 ▸) and Lys110 is conserved in the sequence of *Pd*cyt*c*_552_ (PDB entry 1ql4; Harrenga *et al.*, 2000 ▸) (Supplementary Fig. S5*b* ).

The AioAB/cyt*c*_552_ interaction shows significant charge complementarity, with negative charge on the AioAB complex correlating with a concentration of positive charge on the surface of cyt*c*_552_ (Fig. 2 ▸ *e*). These charged areas encircle neutral surfaces that correlate with the ‘footprints’ of each electron transfer partner on the other. There are between four and 11 water molecules (over the three AioAB/cyt*c*_552_ complexes per asymmetric unit) that sit between AioAB and cyt*c*_552_ and which interact with polar and charged surface residues. Superposition of the coordinates of the AioA and AioB subunits from this work with those of AioAB alone (PDB entry 4aay; Warelow *et al.*, 2013 ▸), yields r.m.s.d. values of 0.20 and 0.28 Å, respectively (over 832 and 132 common C^α^ positions), indicating minimal changes on association with cyt*c*_552_ (Supplementary Fig. S4*b* ).

Within the AioAB/cyt*c*_552_ complex, the cyt*c*_552_ protein shows an average *B* factor of 44.0 Å^2^, which is significantly higher than that of AioAB (23.6 Å). In addition, the relative temperature factor per residue for cyt*c*_552_ increases with increasing distance from the AioAB/cyt*c*_552_ interface (Supplementary Fig. S4*a* ), indicating that the cyt*c*_552_ molecule is dynamic relative to AioAB within the crystalline lattice. This has been observed previously for related electron transfer complexes, such as the SorT/SorU complex from *S. meliloti* (McGrath *et al.*, 2015 ▸). Flexibility in the interactions between protein partners has been proposed to be necessary to achieve optimal orientations for efficient electron transfer (Leys & Scrutton, 2004 ▸; van Amsterdam *et al.*, 2002 ▸).

Taken together, these observations are consistent with the structures of other comparable electron transfer complexes, which typically feature a modest number of hydrogen bonds and salt bridges at the protein–protein interface and relatively small, buried surface areas between protein partners (Supplementary Table S1). The AioAB/cyt*c*_552_ structure is therefore typical of transient complex formation for fast electron transfer (Miyashita *et al.*, 2003 ▸).

### 3.3. The electron transfer pathway between AioAB and cyt*c*_552_

**Figure 3:**
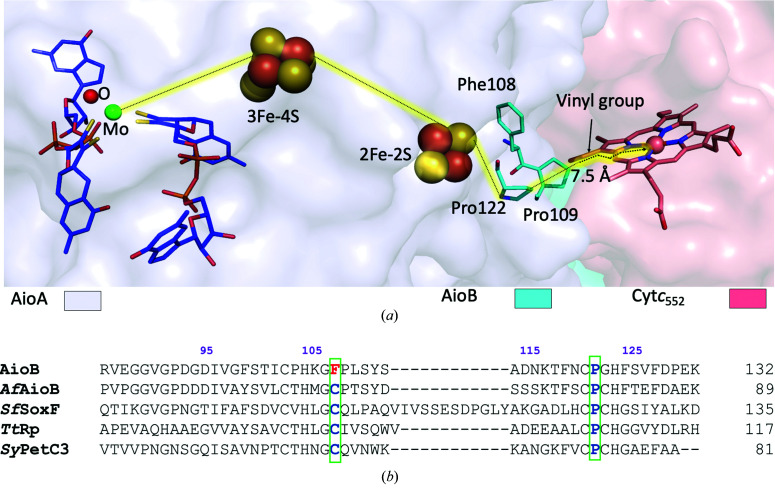
The electron transfer pathway between AioAB and cyt*c*_552_. (*a*) Pathway for electron transfer (Kurnikov, 2000 ▸). (*b*) Secondary-structure-based sequence alignment of Rieske proteins. Conserved residues near the Rieske cluster are colored blue and Phe108, which is unique to AioB, is in red. Residue numbers in purple correspond to the AioB sequence. The alignment was generated using *Clustal Omega* (Sievers *et al.*, 2011 ▸). Abbreviations used are as follows: AioB, *P. banfieldiae* sp. strain NT-26 (this work); *Af*AioB, AioB subunit of arsenite oxidase from *A. faecalis*; *Sf*SoxF, Rieske protein II from *S. acidocaldarius*; *Tt*Rp, Rieske protein from *T. thermophilus*; *Sy*PetC3, Rieske protein from *Synechocystis* PCC 6803.

The vinyl groups on the porphyrin ring of cyt*c*_552_ contribute to the AioAB/cyt*c*_552_ interface. AioB residues Phe108, Pro109 and Pro122 lie closest to the cyt*c*_552_ heme (Fig. 3 ▸ *a*). As mentioned previously, the closest edge-to-edge distance between Cys103, which coordinates the Rieske cluster in AioB, and the vinyl group of heme in cyt*c*_552_ is 7.5 Å, which is well within the distance for fast electron transfer through the protein medium (Page *et al.*, 1999 ▸). *HARLEM* analysis of the coordinates of the complex (Kurnikov, 2000 ▸) predicts that the dominant electron-tunneling pathway from AioB to cyt*c*_552_ proceeds from the [2Fe–2S] Rieske cluster in the AioB subunit to Pro122 and across the protein–protein interface to the porphyrin ring and onto the heme iron, with Pro122 at a distance of 4.1 Å from the closest heme vinyl group (Fig. 3 ▸ *a*, Supplementary Table S2). This suggests a role for Pro122 in the electron transfer process, which is consistent with its conservation in the sequences of Rieske cluster-containing proteins and subunits, including arsenite oxidase from *Alcaligenes faecalis* (PDB entry 1g8k; Ellis *et al.*, 2001 ▸), the Rieske protein II SoxF from *Sulfolobus acido­caldarius* (*Sf*SoxF; PDB entry 1jm1; Bönisch *et al.*, 2002 ▸), the Rieske protein from *Thermus thermophilus* (*Tt*Rp; PDB entry 1nyk; Hunsicker-Wang *et al.*, 2003 ▸) and the Rieske protein involved in photosynthetic and respiratory electron transport in *Synechocystis* PCC 6803 (*Sy*PetC3; PDB entry 5cxm; Veit *et al.*, 2016 ▸; Fig. 3 ▸ *b*). The involvement of proline residues in interprotein electron transfer has previously been proposed for the complex between the nitrite reductase from *Achromobacter xylos­oxidans* GIFU 1051 and its electron acceptor cytochrome *c* (the AxgNIR/cyt*c*_551_ complex; PDB entry 2zon; Nojiri *et al.*, 2009 ▸).

Previous studies examining the kinetics of arsenite oxidation catalyzed by AioAB with horse heart cytochrome *c* (hhcyt*c*) as the electron acceptor revealed that mutation of Phe108 in AioB to Ala led to a 30-fold decrease in the rate of the reaction relative to the native enzyme (Warelow, 2015 ▸). These data are consistent with the observation from the present structure that Phe108 lies at the interface between the AioB and cyt*c*_552_ proteins (the Phe108–heme distance is 5.2 Å). Aromatic residues such as phenylalanine have been shown to be involved in electron transfer in other complexes (Hirasawa *et al.*, 1998 ▸; Liang *et al.*, 1987 ▸), including in the structure of *caa*_3_-type cytochrome oxidase from *T. thermophilus* (Lyons *et al.*, 2012 ▸). Whether this residue is part of the electron transfer pathway to cyt*c*_552_ or facilitates and/or stabilizes complex formation requires further investigation. Notably, this residue is not conserved in the sequences of comparable Rieske proteins (Fig. 3 ▸ *b*).

### 3.4. AioAB is catalytically efficient in the presence of cyt*c*_552_ in solution

Previous studies have reported the kinetics of arsenite oxidation catalyzed by AioAB using the artificial electron acceptors DCPIP and hhcyt*c*. To complement our structural analyses, we determined the activity of the AioAB enzyme with its native electron acceptor cyt*c*_552_. In the presence of an excess of cyt*c*_552_, Michaelis–Menten analysis of arsenite oxidation by AioAB monitored spectrophotometrically yielded a *K*_m(arsenite)_ of 9.06 ± 1.3 µ*M* and a turnover number of 205 ± 19 s^−1^. These values are similar to those reported with hhcyt*c* as the electron acceptor (13.0 ± 0.15 µ*M* and 211.2 ± 0.15 s^−1^, respectively). Analyses in the presence of an excess concentration of arsenite yielded *K*_m(cyt*c*552)_ = 2.9 ± 0.2 µ*M* and *k*_cat_ = 390 ± 25 s^−1^ (Supplementary Table S3).

The similarity of the turnover numbers for arsenite oxidation with cyt*c*_552_ and hhcyt*c* as electron acceptors is interesting given that one protein is a native partner and the other is not. Both proteins show positively charged surfaces and basic pI values (8.7 and 10.0, respectively) and have similar redox potentials [cyt*c*_552_, 275 mV (Kalimuthu *et al.*, 2014 ▸); hhcyt*c*, 256–266 mV (Weber *et al.*, 1987 ▸)]. Presumably, these features allow hhcyt*c* to substitute for cyt*c*_552_ in the *in vitro* assay. The electrostatic surfaces of other *c*-type cytochrome electron acceptors which serve as electron transfer partners to members of the molybdenum protein family also have an overall positive charge. The value of *K*_m(cyt*c*552)_ determined here is similar to those reported for these systems, including chicken liver sulfite oxidase (CSO; *G. gallus*; Kisker *et al.*, 1997 ▸) and the sulfite dehydrogenase SorAB complex from *Starkeya novella*, with *K*_m(cyt*c*)_ values between 2 and 4 µ*M* (Kappler *et al.*, 2006 ▸; Kappler & Enemark, 2015 ▸). A notable exception is the SorT/SorU complex from *S. meliloti* (McGrath *et al.*, 2015 ▸). The electron acceptor SorU has an overall negative charge (pI of ∼4) and a higher *K*_m(SorU)_ (32 ± 5 µ*M*).

The *k*_cat_ values for these systems vary (Supplementary Table S3, with the AioAB/cyt*c*_552_ system apparently being particularly efficient (Brody & Hille, 1999 ▸; Kappler *et al.*, 2006 ▸). The docking and dissociation of AioAB/cyt*c*_552_ before and after electron transfer, respectively, presumably play a significant role in the rate of turnover and can be influenced by the electrostatic complementarity and the number of interactions at the protein–protein interface (Leys & Scrutton, 2004 ▸).

## 4. Conclusion

The structure of the AioAB/cyt*c*_552_ complex reported here shows an interesting combination of ‘functional’ and ‘nonfunctional’ assemblies within the crystals. The positioning of the unique cyt*c*_552_ molecule between AioAB heterodimers presumably facilitates crystallization but does not represent a fast electron transfer complex. The remaining three AioAB/cyt*c*_552_ modules per asymmetric unit show the cyt*c*_552_ molecules positioned in a cleft between the AioA and AioB subunits, with close association between the redox-active cofactors for fast electron transfer.

## 5. Related literature

The following references are cited in the supporting information for this article: Axelrod *et al.* (2002 ▸), Kappler & Bailey (2005 ▸), Krissinel & Henrick (2005 ▸) and Kurisu *et al.* (2001 ▸).

## Supplementary Material

PDB reference: AioAB/cyt*c*_552_ complex, 8ed4

Supplementary Figures and Tables. DOI: 10.1107/S2059798323002103/cb5144sup1.pdf

## Figures and Tables

**Table 1:** Crystallization

Method	Hanging-drop vapor diffusion
Plate type	24-well VDX plates
Temperature (K)	293
Protein concentration (mg ml^−1^)	5
Buffer composition of protein solution	50 m*M* Tris, 100 m*M* NaCl pH 7.8
Composition of reservoir solution	0.2 *M* sodium chloride, 0.1 *M* HEPES pH 7.3, 18%(*w*/*v*) PEG 3350
Volume and ratio of drop	2 µl, 1:1
Volume of reservoir (µl)	500

**Table 2:** Data collection and processing Values in parentheses are for the highest resolution shell.

Diffraction source	MX2, Australian Synchrotron
Wavelength (Å)	0.953
Temperature (K)	100
Detector	EIGER 16M
Crystal-to-detector distance (mm)	249
Total rotation range (°)	360
Space group	*P*2_1_
*a*, *b*, *c* (Å)	129.4, 126.6, 148.0
α, β, γ (°)	90.0, 107.8, 90.0
Mosaicity (°)	0.09
Resolution range (Å)	49.19–2.25 (2.29–2.25)
Total No. of reflections	1510469 (77078)
No. of unique reflections	214836 (10599)
Completeness (%)	99.9 (99.9)
Multiplicity	7.0 (7.3)
〈*I*/σ(*I*)〉	11.4 (2.5)
CC_1/2_	0.998 (0.915)
*R*_merge_	0.112 (0.806)
*R*_p.i.m._	0.045 (0.320)
Overall *B* factor from Wilson plot (Å^2^)	29.5

**Table 3:** Structure refinement Values in parentheses are for the highest resolution shell.

Resolution range (Å)	49.23–2.25 (2.31–2.25)
Completeness (%)	92.2
No. of reflections, working set	188208
No. of reflections, test set	10062
Final *R*_cryst_	0.183 (0.208)
Final *R*_free_	0.230 (0.293)
No. of non-H atoms	
Protein	34093
Waters	1751
Total	35834
R.m.s.d.s	
Bond lengths (Å)	0.007
Angles (°)	1.606
Average *B* factors† (Å^2^)	
Protein	22.9
Waters	9.2
Other	9.9
Ramachandran plot‡	
Most favored (%)	94.2
Allowed (%)	99.8
Outliers§ (%)	0.2
PDB code	8ed4

† Calculated by *BAVERAGE* from the *CCP*4 suite (Winn *et al.*, 2011 ▸).

‡ Calculated using *MolProbity* (Chen *et al.*, 2010 ▸).

§ The outliers were Asp*A*613, Ile*A*811, Asp*C*668, Ile*C*811, Ala*G*304, Ile*G*811, Phe*I*105, Phe*J*105 and Phe*K*105. The 2*F*_o_ − *F*_c_ electron-density maps were observed clearly for these residues at 1.1σ and most are consistent between copies in the asymmetric unit and (for AioAB) with the previously published structure.
