# Supplementary material for: The structure of the complex between the arsenite oxidase from Pseudorhizobium banfieldiae sp. strain NT-26 and its native electron acceptor cytochrome c 552
Source: Acta Crystallogr D Struct Biol. 2023 Mar 30;79(Pt 4):345–52. doi: 10.1107/S2059798323002103 (PMC10071563; doi:10.1107/S2059798323002103)
Supplement: Supplementary file 1 [file d-79-00345-sup1.pdf]

STRUCTURAL BIOLOGY

Volume 79 (2023)

Supporting information for article:

The structure of the complex between the arsenite oxidase from *Pseudorhizobium banfieldiae* sp. strain NT-26 and its native electron acceptor cytochrome c_{552}

Nilakhi Poddar, Joanne M. Santini and Megan J. Maher

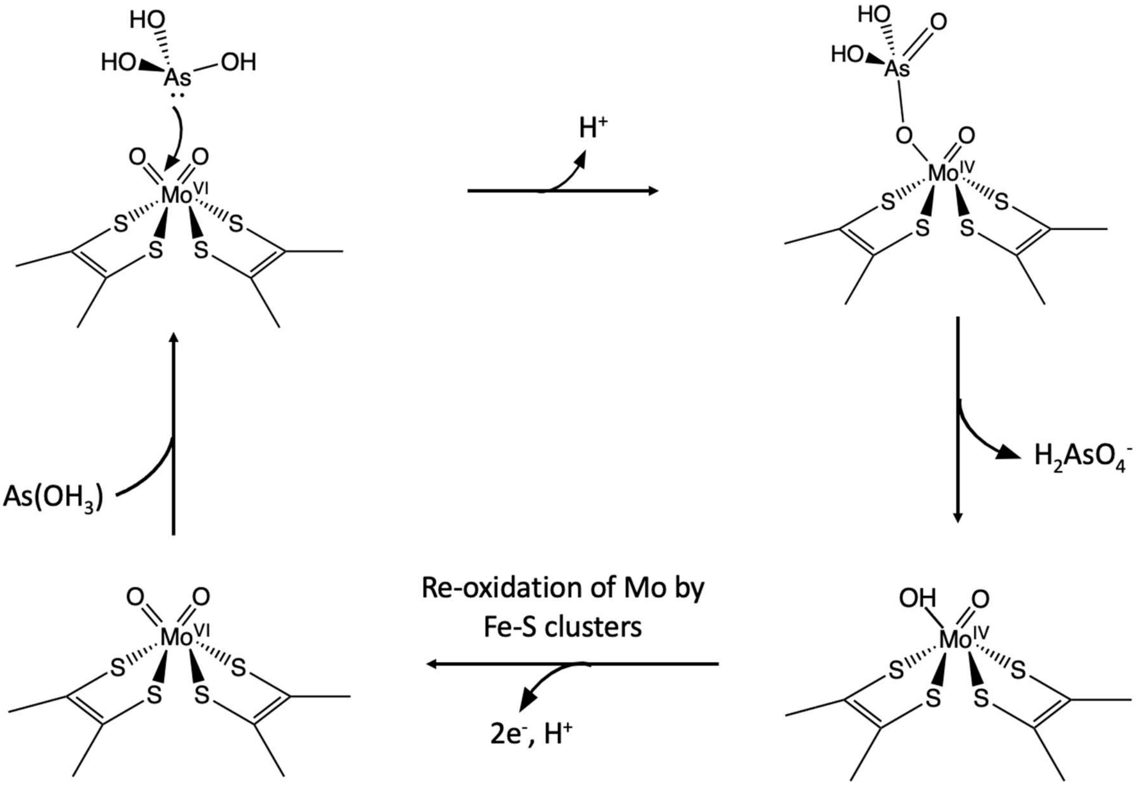

Figure S1 Proposed reaction mechanism of arsenite (As^{III}) oxidation at the Mo active site of the AioA subunit. The As^{III} [As(OH_3)] is oxidised to As^V [H_2AsO_4], reducing Mo^{VI} to Mo^{IV}. The Mo atom is re-oxidised by electron transfer to the Fe-S clusters. Adapted (Kalimuthu *et al.*, 2014).

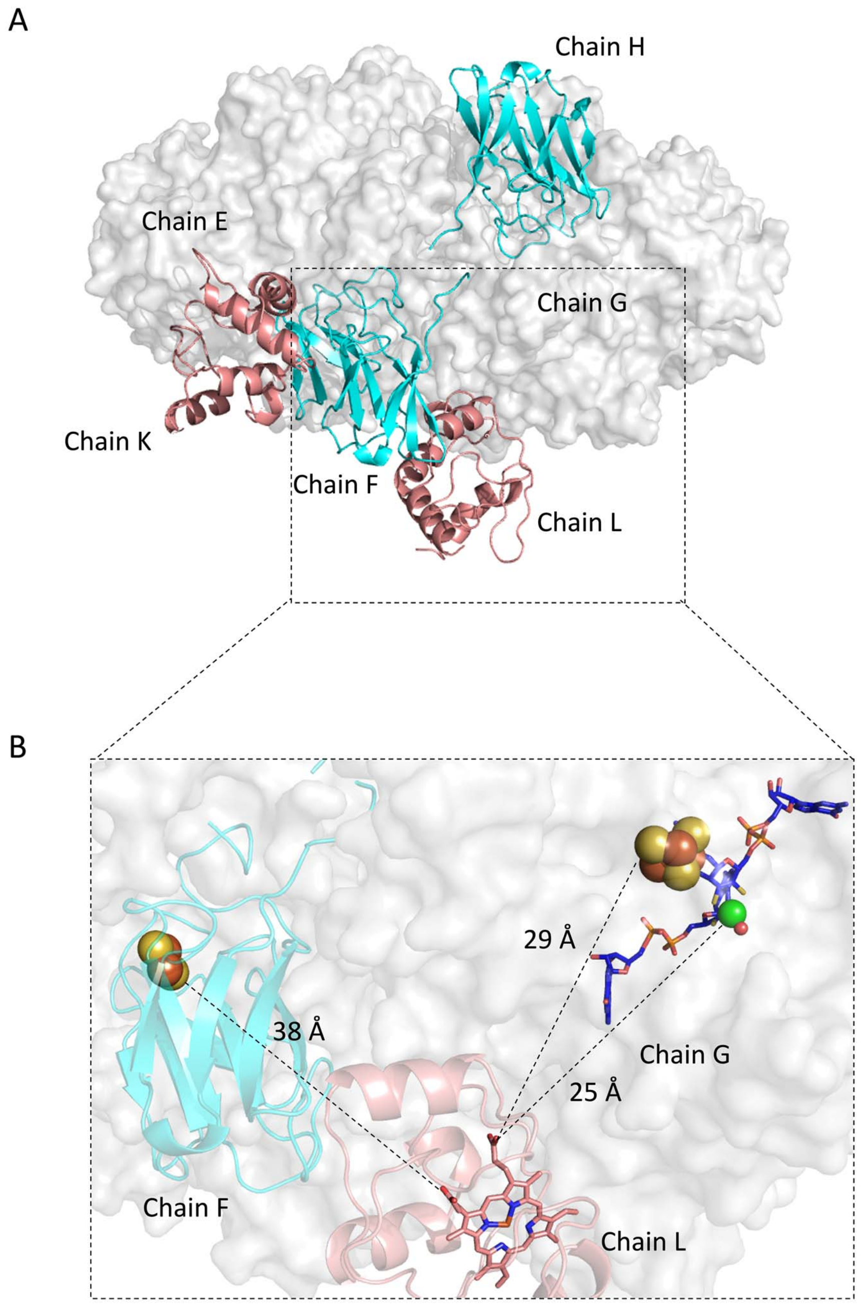

Figure S2 Positioning of the unique cytc_{552} molecule between AioAB heterodimers presumably facilitates crystallization. **(A)** The $AioA_2B_2$ complex is comprised of chains EFK and GHL. The cytc_{552} proteins (chains K and L) are colored in salmon, the AioB subunits (chains H and F) in cyan and the AioA subunits (chains E and G) in gray. **(B)** The Fe-S clusters are shown as yellow and orange spheres and the Moco with oxygen and Mo atoms in red and green, respectively. The edge-edge distances between the cytc_{552} heme, the Moco and the [3Fe-4S] cluster of AioA and the [2Fe-2S] Rieske cluster of AioB are shown.

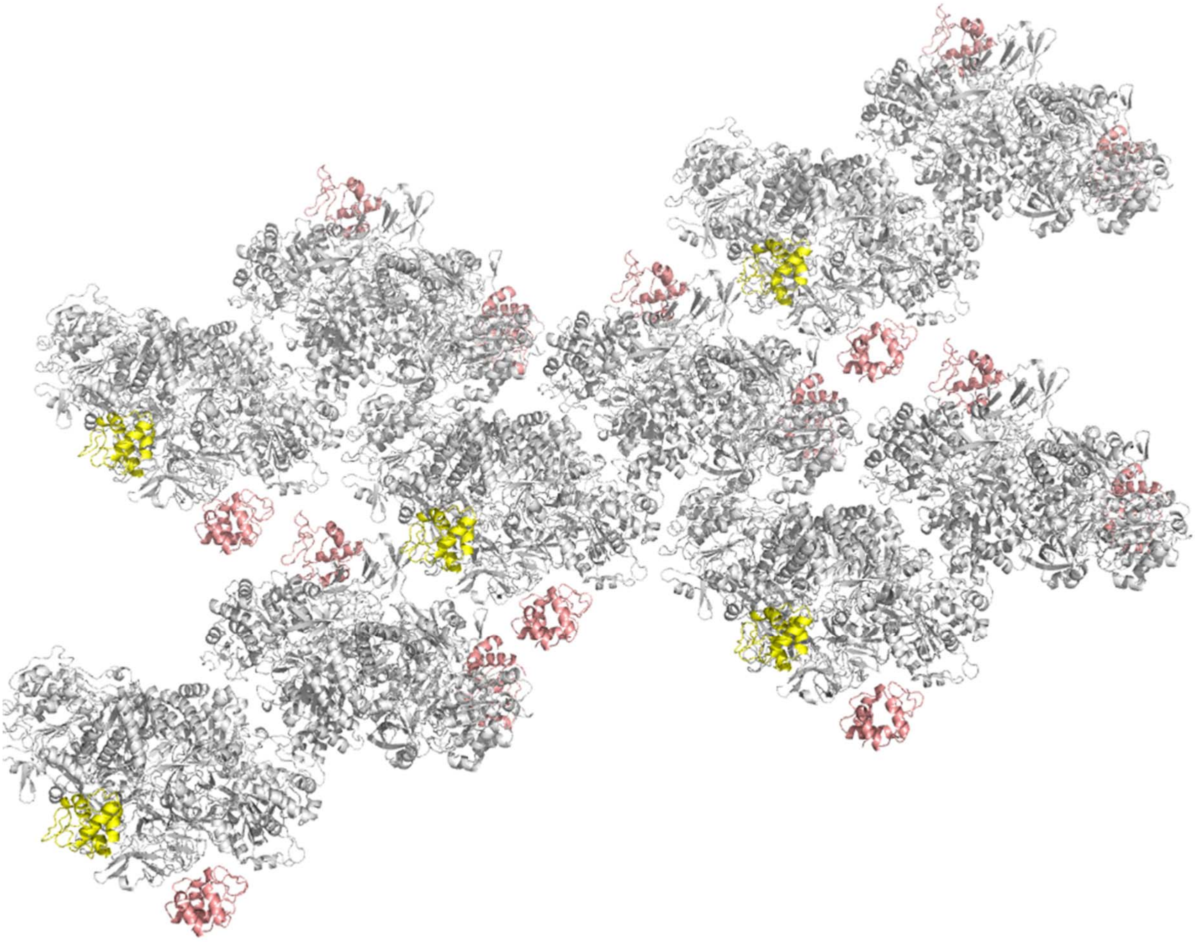

Figure S3 Packing in the AioAB/cytc_{552} crystal. The AioAB heterodimers are colored gray. The cytc_{552} molecules that are positioned at the AioAB interface (part of the 'functional' complex) are shown in salmon, and cytc_{552} molecules that sit between AioAB heterodimers (the 'non-functional' complexes) are colored yellow.

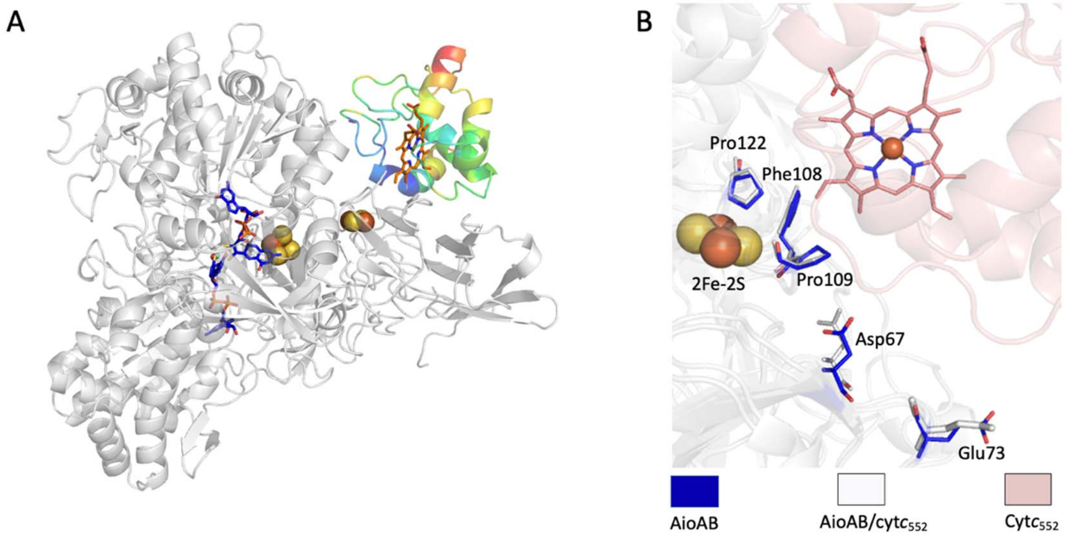

Figure S4 The AioAB/cytc_{552} complex. **(A)** Structure of the AioAB/cytc_{552} complex where the C_α trace of the heme containing cytc_{552} is colored according to temperature factor (from blue ('low' *B*) to red ('high' *B*)). **(B)** Superposition of the coordinates of AioAB from the present structure, with that of the AioAB protein alone (PDB 4AAY) (Warelow *et al.*, 2013). Residues at the AioAB/cytc_{552} interface show no major conformational changes upon complex formation. The [2Fe-2S] Rieske cluster is shown as yellow and orange spheres. AioAB and cytc_{552} are represented in gray and salmon, respectively and the superposed structure of AioAB alone is blue.

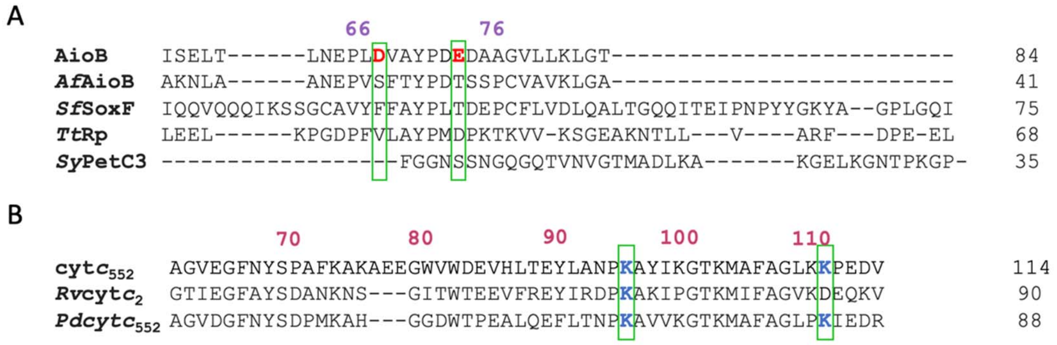

Figure S5 Secondary structure-based sequence alignment of Rieske protein and cytochrome *c* sequences related to AioAB/cytc_{552}. **(A)** Interface residues Asp67 and Glu73 are unique to AioB (red). Residue numbers for AioB are represented in purple. **(B)** Conserved residues between the sequence of cytc_{552} are colored blue. Residue numbers for cytc_{552} are shown in red. The alignment was generated using ClustalW Omega (Sievers *et al.*, 2011). Abbreviations used are as follows: AioB, *Pseudorhizobium banfieldiae* sp. str. NT-26 (this work); *Af*AioB, AioB subunit of arsenite oxidase from *Alcaligenes faecalis*; *Sf*SoxF, Rieske protein II from *Sulfolobus acidocaldarius*; *Tt*Rp, Rieske protein from *Thermus thermophilus*; *Sy*PetC3, Rieske protein from *Synechocystis* PCC 6803; cytc_{552}, cytochrome c_{552} from *Pseudorhizobium banfieldiae* sp. str. NT-26 (this work); *Rv*cytc_2, cytochrome c2 from *Rhodopseudomonas viridis*; *Pd*cytc_{552}, cytochrome c_{552} from *Paracoccus denitrificans*.

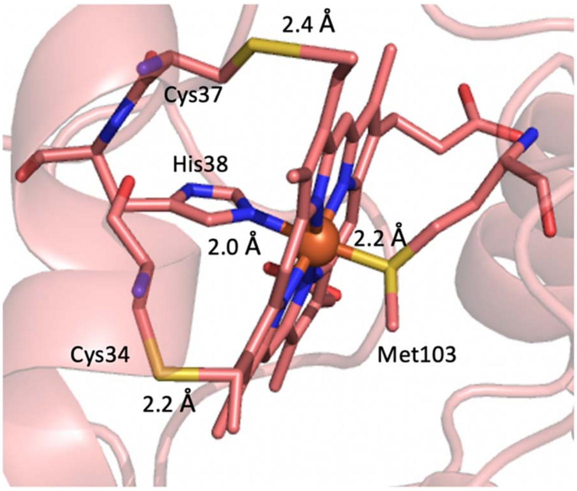

Figure S6 The heme binding site in cytc_{552}. The heme iron atom is coordinate by residues His38 and Met103. The protoporphyrin ring is covalently attached to residues Cys34 and Cys37.

Table S1 Comparison of the protein-protein interfaces from AioAB/cytc_{552} and related electron transfer complexes.

**Structures**	**AioAB/cytc_{552}**[a]		**SorT/SorU**[b]		**AxgNIR/Cytc_{551}**[c]		**FNR/Fd**[d]		**Cytochrome c_2: reaction center**[e]	
Parameter	AioAB	cytc_{552}	Sor T	SorU	AxgNIR	Cytc_{551}	FNR	Fd	Cytochrome c_2	Reaction center
Buried surface area (Å^2)[f]	680	660	644	696	529	553	800	800	674	614
Interfacing residues[g]	25	18	31	21	10	11	25	21	21	23
Edge to edge distance between redox centres (Å)	7.5		8.2		10.5		6		8.4	
Hydrogen bonds	0		6		3		0		3	
Salt-bridges	2		1		0		5		0	
PDB ID	8ED4		4PW9		2ZON		1GAW		1L9J	

[a] This work.

[b] PDB code 4PW9 (McGrath *et al.*, 2015).

[c] PDB code 2ZON (Nojiri *et al.*, 2009).

[d] PDB code 1GAW (Kurisu *et al.*, 2001).

[e] PDB code 1L9J (Axelrod *et al.*, 2002).

[f] Calculated as the average for the promoter of interest divided by the average for the entire complex structure.

[g] Calculated by using PISA (Krissinel & Henrick, 2005).

Table S2 Electron transfer parameters between AioB ([2Fe-2S]) and cytochrome c_{552} (heme)[a].

Atomic packing density (ρ)	0.69
Distance (Pro122 in AioB subunit – Heme) (Å)	4.1
Average decay exponent (β)	1.4
Electron coupling (H_{DA})	1.3 X 10^{-3}
Maximum ET rate (s^{-1})	1.6 X 10^{8}

[a] Calculated using HARLEM (Kurnikov, 2000).

Table S3 Kinetic parameters of enzymes determined with their native electron acceptors.

**Enzyme**	**Electron acceptor**	$K_{M(cytc)}$ (μM)	K_{cat} (s^{-1})	K_{cat}/K_M ($M^{-1}.s^{-1}$)
AioAB [a]	Cytochrome c_{552}	2.9 ± 0.2	390 ± 25	135.4 x 10^6
SorT [b]	SorU	32 ± 5	140 ± 11	4.3 x 10^6
Chicken sulfite oxidase [c]	cytochrome *c*	2.2 ± 0.05	95.0 ± 1.9	43.2 x 10^6
SorAB [d]	cytochrome c_{550}	4.0	334 ± 11	8.3 x 10^6

[a] This work.

[b] (McGrath *et al.*, 2015).

[c] (Brody & Hille, 1999)

[d] (Kappler & Bailey, 2005).

Table S4 Complete amino-acid sequences of AioA, AioB and cytc_{552} .

Protein	
AioA subunit	AFKRHIDRLPIIPADAKKHNVTCHFCIVGCGYHAYTWPINKQGGTDPQNNIFGVDLSEQ<br>QQAESDAWYSPSMYNVVKQDGRDVHVVIKPDHECVVNSGLGSVRGARMAETSFSEA<br>RNTQQQRLTDPLVWRYGQMQPTSWDDALDLVARVTAKIVKEKGEDALIVSAFDHGG<br>AGGGYENTWGTGKLYFEAMKVKNIRIHNRPAYNSEVHGTRDMGVGELNNCYEDAEL<br>ADTIVAVGTNALETQTNYFLNHWIPNLRGESLGKKKELMPEEPHEAGRIIIVDPRRTVT<br>VNACEQTAGADNVLHLAINSGTDLALFNALFTYIADKGWVDRDFIDKSTLREGTARPP<br>LYPARGVSEANPGHLSSFEDAVEGCRMSIEEAAEITGLDAAQIIKAAEWIGMPKEGGKR<br>RRVMFGYEKGLIWGNDNYRTNGALVNLALATGNIGRPGGGVVRLGGHQEGYVRPSD<br>AHVGRPAAYVDQLLIGGQGGVHHIWGCDHYKTTLNAHEFKRVYKKRTDMVKDAMS<br>AAPYGDREAMVNAIVDAINQGGLFAVNVDIIPTKIGEACHVILPAATSGEMNLTSMNG<br>ERRMRLTERYMDPPGQSMPDCLIAARLANTMERVLTEMGDVGYAAQFKGFDWQTEE<br>DAFMDGYNKNAHGGEFVTYERLSAMGTNGFQEPATGFTDGKIEGTQRLYTDGVFSTD<br>DGKARFMDAPWRGLQAPGKQQQKDSHKYLINNGRANVVWQSAYLDQENDFVMDRF<br>PYPFIEMNPEDMAEAGLKEGDLVEIYNDAGATQAMAYPTPTARRGETFMLFGFPTGV<br>QGNVTSAGTNELIIPNYKQTWGNIRKISDAPRNVAHLSFKSKEYQSA
AioB subunit	**HHHHHHDYDIPTTENLYFQGAMGSGIQ**ATAAAGVEYPANRLANISELTLNEPLDVA<br>YPDEDAAGVLLKLGTRVEGGVGPDGDIVGFSTICPHKGFPLSYSADNKTFNCPGHFSVF<br>DPEKGGQQVWGQATQNLPQYVLRVADNGDIFAEGVDELIYGRLSNVL
cytc_{552}	**MD**ESNAEKGAVVFKKCAACHAVGDGAANKVGPELNGLIGRKVAGVEGFNYSPAFKA<br>KAEEGWVWDEVHLTEYLANPKAYIKGTKMAFAGLKKPEDVADVIAYLKTFSTP**LEH**<br>**HHHHH**

[a]Bold and underlined amino-acid residues are derived from the affinity tags and cleavage sites.
